# Validation of the Revised Olweus Bully/Victim Questionnaire (OBVQ-R) Among Adolescents in Chile

**DOI:** 10.3389/fpsyg.2021.578661

**Published:** 2021-04-12

**Authors:** Jorge Gaete, Daniela Valenzuela, María Inés Godoy, Cristian A. Rojas-Barahona, Christina Salmivalli, Ricardo Araya

**Affiliations:** ^1^Faculty of Education, Universidad de los Andes, Santiago, Chile; ^2^Millennium Nucleus to Improve the Mental Health of Adolescents and Youths, Imhay, Santiago, Chile; ^3^Departamento de Evaluación, Medición y Registro Educacional, Universidad de Chile, Santiago, Chile; ^4^Faculty of Psychology, Universidad de Talca, Talca, Chile; ^5^School of Psychology, University of Turku, Turku, Finland; ^6^Department of Health Service and Population Research, King’s College London, David Goldberg Centre, London, United Kingdom

**Keywords:** validity, reliability, bullying, OBVQ-R, questionnaire, children, adolescents, Chile

## Abstract

Bullying is a phenomenon that affects children and adolescents worldwide, and it has major consequences for all participants involved in these situations. In Chile, researchers have validated several instruments used to investigate aggression between peers and school violence, but there is a lack of validation of instruments to investigate bullying. The purpose of this study was to provide evidence of the validity and reliability of the Olweus Bully/Victim Questionnaire—Revised version (OBVQ-R) in the Chilean context. The participants were 2,775 students from schools of low, medium, and high socioeconomic status. OBVQ-R is a self-report questionnaire with 42 items, which has been used in different countries, and has adequate psychometric properties to assess the prevalence of victimization and aggression and various forms of bullying worldwide. Results confirmed the two-factor structure of the OBVQ-R (victimization and perpetration subscales) and good reliability (ω = 0.81 and ω = 0.75, respectively). These dimensions seem to be correlated. Comparison between OBVQ-R with the School Violence between Peers Questionnaire and the Internet Experiences Questionnaire showed some degree of agreement. The Item Response Theory analysis showed that the item about verbal bullying, in both subscales, had the lowest-severity parameters, meaning that these forms of bullying were the most prevalent. The higher-severity parameter in the victimization scale was the cyberbullying item, and the sexual bullying item showed higher severity in the perpetration subscale. The differential item functioning analysis by gender showed a trend in which boys responded with lower-severity parameters than girls. In the victimization scale, the exception was the item about spreading rumors, and in the perpetration subscale, it was the item about racial bullying. We have provided evidence of the validation of OBVQ-R among school-age children and adolescents in Chile. This study is part of a trial registered at ClinicalTrials.gov, number NCT02898324.

## Introduction

Bullying is a major educational and public health problem (Menesini and Salmivalli, 2017). Bullying has been defined as intentional aggressive behaviors that repeatedly occur over time and in the context of a power imbalance between the victim/s and the perpetrator/s (Olweus, 1978; Menesini and Salmivalli, 2017). Bullying behavior may take different forms, such as physical (e.g., hitting, kicking, pushing), verbal (e.g., insults and telling mean names), and social or relational aggression (e.g., social exclusion, spreading rumors, or online attacks) (National Academies of Sciences, Engineering, and Medicine, 2016).

Studies show that the prevalence of bullying varies across countries and studies. This may be due to the use of different instruments, and the definitions and operationalization of the bullying concept (Menesini and Salmivalli, 2017). Nonetheless, the prevalence rates are high. For example, in a recent systematic review of studies conducted in Australia, the lifetime prevalence of bullying victimization was 25.1% and perpetration was 11.6%. For cyberbullying, the estimates were less common, 7% for victimization and 3.5% for perpetration (Jadambaa et al., 2019). Another recent meta-analysis of youth between 12 and 18 years old (*n* = 335,519) showed that 35% of students were involved in traditional bullying and 15% in cyberbullying (Modecki et al., 2014). Very few studies have been carried out in Latin-American countries. For instance, in Brazil, in a study conducted among 60,973 students exploring a 30-day prevalence of bullying, 5.4% reported that they had been continually bullied and 25.4% reported rarely being bullied (Malta et al., 2010). In Argentina, Resett (2016) found the following prevalence: victims 13%, bullies 6%, bully/victims 5%, and non-involved students 73%.

Regarding gender, differences between boys and girls in traditional bullying appear to be consistent. For instance, males seem to be more frequent perpetrators and victims in traditional bullying (Smith et al., 2019). However, there is a considerable variation between countries (Smith et al., 2019). Less consistent results appear in cyberbullying, where some studies report no gender differences (Brown et al., 2014), while others have found a higher proportion of females as victims (Kowalski and Limber, 2007).

There is a less clear association between socioeconomic status (SES) and bullying. In a recent systematic review and meta-analysis including 28 studies, mostly from Europe and North America, victimization and bully-victim status were positively but weakly associated with low SES, while bullying perpetration was the most weakly related (Tippett and Wolke, 2014). Further research exploring this association in middle- and low-income countries is needed.

Bullying has negative consequences in all children and adolescents involved, and some of these effects can last until adulthood (Salmivalli and Peets, 2018). For instance, victims of bullying exhibit more depressive, anxious, and somatic symptoms, lower self-esteem, lower academic performance, and suicidal ideation, among other problems (Skapinakis et al., 2011; Heerde and Hemphill, 2019). On the other hand, bullies have a higher risk for externalizing symptoms such as delinquent behaviors, substance misuse, impulsive behavior, and lower anger regulation compared with non-perpetrator students (Haynie et al., 2001). Children and adolescents who identified themselves as bully victims share the psychological consequences of both groups, and research has shown that they are the most maladjusted group (Haynie et al., 2001; Rivers, 2011). Some studies show that there are also negative consequences for bystanders, such as a higher risk of substance use than students not involved in bullying situations (Polanin et al., 2012; Gaete et al., 2017).

There are some gender differences regarding the consequences of bullying. Consistent results have been found regarding female victims having a higher risk for internalizing symptoms such depression and suicidal ideation (Fredrick and Demaray, 2018; Cao et al., 2020). Similarly, girls report higher negative psychological symptoms and suicidal behavior than boys (Rey et al., 2019). On the other hand, externalizing problems after being bullied are also present, especially among boys (McDougall and Vaillancourt, 2015).

### Bullying Assessment

The self-report questionnaire is the most common methodology used for data collection to assess bullying (Ronan et al., 2013; Nelson et al., 2018). This method has several advantages: it takes a relatively short time to administer, is generally easy to answer, is relatively inexpensive (e.g., one evaluator can assess many students), and is efficient (e.g., many students can be evaluated at the same time). Additionally, the same questionnaire can be used by different research teams internationally, therefore allowing cross-country comparisons of prevalence and associated factors (Solberg and Olweus, 2003; Crothers and Levinson, 2004). However, there are also some disadvantages: students may give biased responses to maintain social desirability, especially among perpetrators who can underreport their behavior. It is also challenging to obtain in-depth information about bullying experiences, especially when compared with data gathered by interviews (Crothers and Levinson, 2004). Nonetheless, self-reported questionnaires are still the best option for assessing large populations and evaluating the effectiveness of bullying prevention programs.

Additionally, Evans et al. (2014) reviewed 32 articles about the effectiveness of bullying programs and stressed the importance of using an explicit definition of bullying to help responders to have a clear and shared concept of bullying, distinguishing it from other forms of aggression.

### The Olweus Bully/Victim Questionnaire

The Olweus Bully/Victim Questionnaire (OBVQ) is one of the most widely used instruments to measure the prevalence of bullying worldwide (Green et al., 2013; Smith et al., 2016). This questionnaire provides students with a clear definition of bullying that includes the three essential characteristics: (1) intent to cause harm to another person; (2) repetitive conduct; and (3) power imbalance between the victim and the perpetrator (Olweus, 1978; Salmivalli and Peets, 2018). The original version of the OBVQ was developed in 1983 (with 36 items), and in 1996 Dan Olweus put forward the revised questionnaire (OBVQ-R) and increased the number of items to 42 (Kyriakides et al., 2006). Additional questions about different bullying types were included in the revised version, such as sexual bullying and cyberbullying. Additionally, the revised version has a more specific criterion of frequency: the response option “sometimes” in the original version was changed to “2 or 3 times a month” (Solberg and Olweus, 2003).

Several studies have shown evidence of the validity of the OBVQ using different methodological approaches (Solberg and Olweus, 2003; Vessey et al., 2014; Breivik and Olweus, 2015). For instance, Kyriakides et al. (2006) studied the OBVQ-R using Rash modeling in a sample of 335 Greek Cypriot students. The results supported the validity and reliability of the OBVQ-R, showing that there are two main factors (being bullied and bullying others) and that it addresses the typology of indirect, verbal, and physical bullying. Bevans et al. (2013) evaluated the OBVQ victimization scale using Item Response Theory (IRT) in a sample of 17,198 United States students. They found that the questionnaire has a reliable scale (Cronbach’s alpha full scale 0.84) using ten items from the victim dimension. The researchers also concluded that the victimization factor has two subdimensions (direct and indirect types of victimization), and it has a better fit when stratifying by gender. Breivik and Olweus (2015) used IRT modeling and studied the psychometric properties of the OBVQ in a sample of 48,926 students in Norway. They found an optimal scale using eight items (they did not include the item about cyberbullying nor the item about *other forms* of bullying) in which bullying others corresponds to one factor, and the items that had the highest-severity parameters were taking money from others, spreading false rumors, and threatening others.

In Latin America, the OBVQ-R has been used in a few studies, showing satisfactory psychometric properties. In Argentina, two studies have evaluated the psychometric properties of the OBVQ-R. Resett (2011) administered the OBVQ-R to 84 Argentinean students to assess internal consistency. In the victim subscale, the Cronbach’s alpha for the victimization scale was 0.9, with the cyberbullying item having a relatively low item-total correlation (*r* = 0.15). The perpetration scale had a Cronbach’s alpha of 0.81, and the lowest item-total correlation was for threatening others (*r* = 0.16). In another study with 1,222 Argentinean students, a good fit for the two-factor model of the OBVQ-R (being bullied and bullying others) using confirmatory factorial analysis (CFA) was found (Resett et al., 2015). Additionally, the study found differences in the perpetrator subscale between genders, observing that boys identified themselves as harming others by physical aggression, and girls by relational aggression (e.g., lying, spreading rumors). Gonçalves et al. (2016) studied the OBVQ-R in a sample of 713 Brazilian students, from 5th to 9th grade, using IRT modeling. The study showed satisfactory reliability for the victim subscale (Cronbach’s alpha 0.85) and the perpetrator subscale (Cronbach’s alpha 0.87). In this study, the IRT model showed that the direct forms of bullying (e.g., threats, hurtful comments) had a high power to distinguish between victims and bullies. Finally, in another study with 409 Brazilian students, researchers found good internal consistency of the OBVQ (Cronbach’s alpha 0.75) (Zequinão et al., 2016).

No previous study has assessed the validity and reliability of the OBVQ-R in Chile. Having a validated instrument will help to determine the prevalence of bullying, allow cross-country comparisons, and evaluate preventive school-based interventions. We used the Spanish version of the 42-item OBVQ-R.

This study aimed to determine the validity and reliability of the OBVQ-R in a sample of adolescents in Chile. The specific objectives were (1) to study the dimensionality and reliability of the Spanish version of the OBVQ-R in Chilean students; (2) to describe the psychometric features of the questionnaire; (3) to study the concurrent validity of this scale, comparing the OBVQ-R with the School Violence between Peers Questionnaire (MIAP) and Internet Experiences Questionnaire; (4) to study the internal structure of the OBVQ-R using the IRT Rach Model; and (5) to analyze the differential item functioning regarding gender and socioeconomic status.

## Materials and Methods

### Study Design and Participants

This study was an analytical cross-sectional survey using self-reported information. The participants were students attending 4th to 8th grades, in mixed schools located in two central regions in Chile: Metropolitan and Valparaíso regions. The students were between 9 and 16 years old (mean 11.5, SD = 1.6), and 57.1% were female. See Table 1.

**TABLE 1:** Descriptive Variables.

Variables	*n*	% or Mean	[95%IC] or (SD)		
**Gender**					
Female	1582	57.1	[55.3	-	59.0]
Male	1187	42.9	[41.0	-	44.7]
**Class Level**					
4th	538	19.4	[17.9	-	20.9]
5th	545	19.6	[18.2	-	21.1]
6th	551	19.9	[18.4	-	21.3]
7th	572	20.6	[19.1	-	22.1]
8th	569	20.5	[19.0	-	22.0]
**SES**					
Low	773	27.9	[26.2	-	29.5]
Medium	967	34.8	[33.1	-	36.6]
High	1035	37.3	[35.5	-	39.1]
**Type of school**					
Public	488	17.6	[16.2	-	19.0]
Subsidized	1252	45.1	[43.3	-	47.0]
Private	1035	37.3	[35.5	-	39.1]
**Age by class level**					
4th	534	9.4	(0.6)		
5th	543	10.1	(0.6)		
6th	548	11.4	(0.6)		
7th	570	12.4	(0.6)		
8th	568	13.4	(0.7)		

Considering differences according to household incomes (OECD, 2015), 32 schools were invited to participate, representing high, medium, and low SES. The SES was obtained from the Education Quality Measurement System (*Sistema de Medición que la Agencia de Calidad de la Educación*, SIMCE), a measure used in Chile to evaluate different aspects of the school curricula and sociodemographic information from students and their families. Nine of the schools agreed to participate: three of low SES (representing 37.3% of students), four of Medium SES (34.8% of students), and two of high SES (27.9% of students).

### Procedure

The research team obtained authorization from the schools’ authorities. Then, the team asked for informed and written consent from parents/main caregivers. A total of 3,363 parents/main caregivers were contacted, and 99.1% (*N* = 3060) of them consented to their children’s participation. The Ethical Committee of the University of the Andes of Chile approved the study protocol (January 18th, 2016). The study followed the Helsinki Convention norms.

The study was undertaken between June and August 2016, including the recruitment of schools and the evaluation of the students. The questionnaire was administered to the whole class, in the classroom, or in another suitable place in school. Trained research assistants carried out the administration on two different days, 1 week apart, for each class during the school hours (60 min each): on the first day, the students responded to the OBVQ-R, and 1 week later, they answered the MIAP Questionnaire and the Internet Experiences Questionnaire (IEQ).

Before administering the questionnaire, a research assistant asked the students to sign an informed assent to ensure voluntary participation, and a total of 2,775 students agreed to participate. Then, the assistant asked the students to fill in their sociodemographic information. In all grades, the research assistant read out the definition of bullying. In the 4th and 5th grades, the whole questionnaire was read out by the research assistant, but for 6th to 8th grades, the students answered it independently. The research assistants responded to doubts and questions from students. After the students completed the questionnaire, the research assistant put the returned questionnaires in sealed envelopes to ensure confidentiality. Research assistants entered the data in a predesigned database using computers exclusively dedicated to the research. Once the data were entered, the research field coordinator reviewed and cleaned the data. Finally, all personal information (name, school, class) was codified and encrypted, producing a final database without personal information. Only the principal investigator (JG) had access to the data using a password. This procedure allowed us to guarantee confidentiality and anonymity.

### Measures

#### Sociodemographic Variables

The following sociodemographic variables were included in the analysis: gender, school grade, type of dependence, and SES of the educational establishment.

#### Administering the Revised Olweus Bully/Victim Questionnaire

The OBVQ-R is a 42-item self-report questionnaire that assesses events related to bullying behaviors between peers at school using a referential period of 2 months. It also includes questions about attitudes toward bullying and school climate (Solberg and Olweus, 2003) (see Supplementary Material).

Before administering the questionnaire, a definition of bullying was read out aloud for students. After general demographic questions (gender, school grade), the questionnaire started with two global questions where students could identify themselves as victims or bullies: “How often have you been bullied at school in the past couple of months?” (victims), and a similar question for harming others (perpetrators). It also asked about nine types of bullying, which included (1) calling mean names or teasing; (2) exclusion; (3) hitting, kicking, and pushing; (4) spreading rumors; (5) taking money or damaging belongings; (6) threatening; (7) making racial comments; (8) making sexual remarks or gestures; and (9) cyberbullying. It also asked if the children or adolescents had suffered any other form of bullying that was not mentioned (Solberg and Olweus, 2003). Another group of nine questions asked about characteristics of the bullying situations (e.g., the bullies’ grade level, number of bullies, the length of time the student has been suffering from bullying, and the location where it took place). The questionnaire also included nine questions about actions that have been taken in school by teachers or parents to stop bullying. Finally, two questions asked about what students think of teachers’ and parents’ opinions about bullying (Solberg and Olweus, 2003; Vessey et al., 2014).

The answers were coded into a five-point scale from 0 to 4 (0 = *it hasn’t happened to me in the last two months*, 1 = *it happened to me only once or twice in the last two months*, 2 = *it happened to me 2 to 3 times a month*, 3 = *it happened to me once a week*, 4 = *it happened to me several times a week*) (Solberg and Olweus, 2003). The psychometric properties of the OBVQ-R have been studied elsewhere, as mentioned above, and internal consistencies range from 0.8 to 0.9 (Breivik and Olweus, 2015).

#### Violence Between Peers

MIAP was used to assess aggressive behaviors among students (Lecannelier et al., 2011). This questionnaire is a self-report instrument containing 13 multiple-choice questions. The questions gather information about aggression and its frequency using a 4-point scale: 1 = *never*, 2 = *sometimes*, 3 = *often*, and 4 = *always*. This instrument was adapted and validated for the Chilean population by Lecannelier et al. (2011), showing an overall Cronbach’s alpha of 0.89. It distinguishes different roles, such as victim, bully, bully/victim, and bystander. This scale does not assess explicit bullying nor does it use a definition of bullying; however, it does evaluate violence in the schools, identifying several roles. The answers from this questionnaire and the OBVQ-R should be similar to assess the concurrent validity of the OBVQ-R. In our sample, the Cronbach’s alpha was 0.94, and the ω was 0.89 for the victimization factor, and the Cronbach’s alpha was 0.95, and the ω was 0.82 for the perpetration factor.

#### Cyberbullying

The IEQ is a self-report questionnaire with 28 questions. It evaluates different forms of traditional bullying and cyberbullying happening during the current school year. The questionnaire assesses if the respondent was a victim or a bully, the number of times bullying took place, and if the victim knew who was responsible (Raskauskas and Stoltz, 2007). This instrument was adapted and validated for the Chilean population, showing a Cronbach’s alpha of 0.62 for the full scale (Lecannelier et al., 2010). There were three questions closely related to the cyberbullying item included in the OBVQ-R: Have you been a victim of bullying through text messages (using cellphone or WhatsApp)?; Have you been a victim of bullying through internet (messages posted on a website or blog); and Have you been a victim of bullying through using pictures or videos of you without your permission? Similar questions were constructed for evaluating aggression. All these questions were answered using a 6-point scale: 0 = *never*, 1 = *once or twice a year*, 2 = *3–5 times a year*, 3 = *6–10 times a year*, 4 = *11–15 times a year*, and 5 = *16 or more times a year.* It was decided to compare the cyberbullying item included in the OBVQ-R with the question in the IEQ instrument for which the same student had the highest score. We assessed the concurrent validity of the OBVQ-R with these questions.

### Data Analysis

The descriptive statistics are reported as percentages with 95% confidence intervals (CI 95%) for gender, class level, socioeconomic status, type of school, and age reported as mean (standard deviation) by class level.

#### Dimensionality and Reliability

**FIGURE 1:**
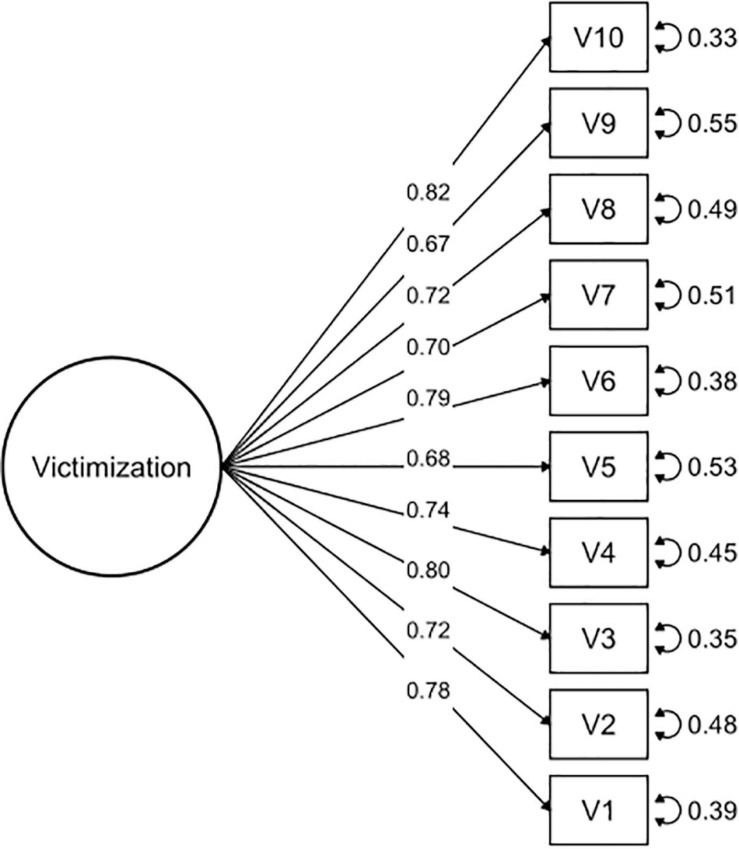
Confirmatory factor analysis of the victimization subscale.

**FIGURE 2:**
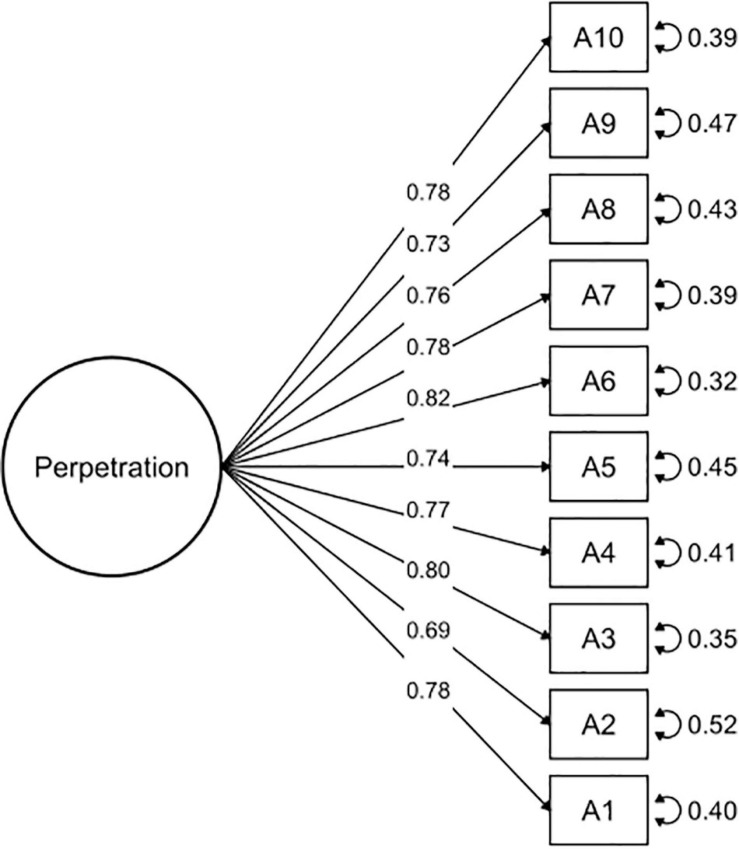
Confirmatory factor analysis of the prepetration subscale.

**FIGURE 3:**
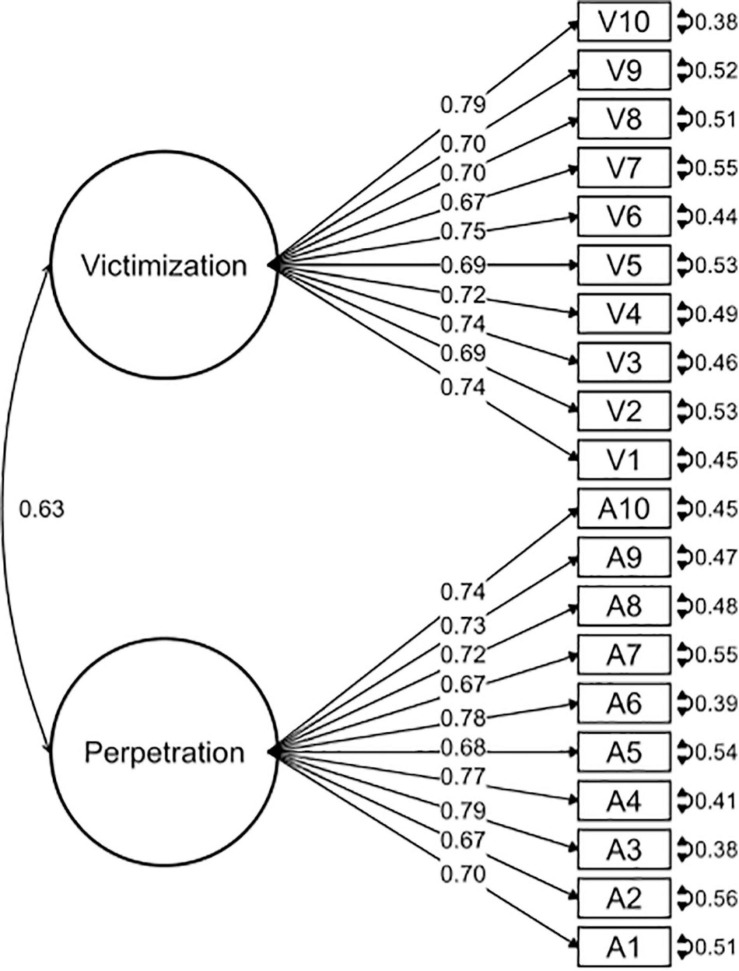
Confirmatory factor analysis of the model with two factors correlated for victimization and perpetration subscales.

**FIGURE 4:**
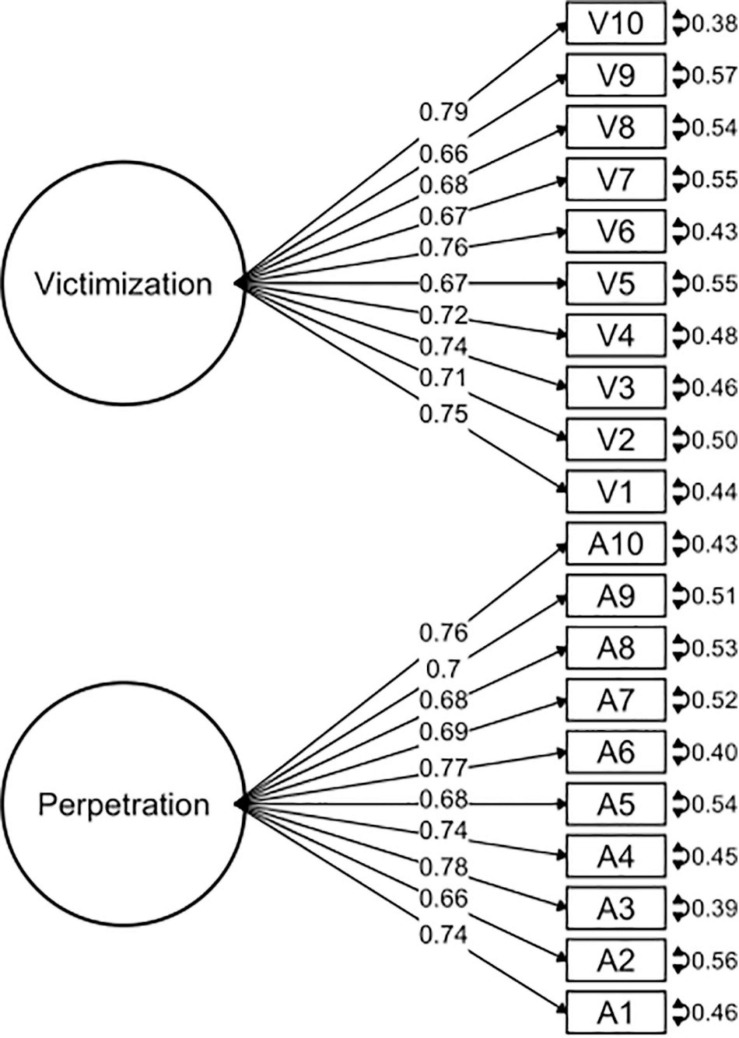
Confirmatory factor analysis of the model with two factors uncorrelated for victimization and perpetration subscales.

A CFA was conducted to study the dimensionality and reliability of the OBVQ-R. We used the weighted least squares (WLS) of the polychoric matrix, which is considered more robust than other methods (Browne, 1984; Muthén, 1984). Polychoric correlations are advised for factor analysis when the distributions of items are ordinals (Flora and Curran, 2004). CFA is part of the measurement model that examines relationships between variables and the observed factors. It was important to determine if the questionnaire had two subscales (victimization and perpetration) and if these subscales were independent of each other (uncorrelated) or were related. Therefore, we assessed the goodness of fit of different models: (1) two independent models of victimization and perpetration subscales (see Figures 1, 2), (2) a model with two factors correlated for victimization and perpetration subscales (see Figure 3), and (3) a model with two factors uncorrelated for victimization and perpetration subscales (see Figure 4). We used multiple goodness-of-fit indices to judge whether the proposed model is consistent with the empirical data, and we used the chi-square test (CHISQ) to compare both models. The following indices were calculated to determine if the adjustment was at least acceptable: (1) Root Mean Square Error of Approximation (RMSEA), (2) Standardized Root Mean Square Residual (SRMR), (3) Normed Fit Index (NFI), (4) Non-normed Fit Index (NNFI), (5) Comparative Fit Index (CFI), (6) Goodness-of-Fit Index (GFI), and (7) Adjusted Goodness-of-Fit Index (AGFI) (Table 2). It was considered a good fit for RMSEA if values were less than or equal to 0.05, and values between 0.05 and 0.08 were considered adequate (Bollen and Long, 1993). SRMR values less than 0.05 indicated a good fit, while values less than 0.10 were interpreted as acceptable (Hu and Bentler, 1995). NFI greater than or equal to 0.95 indicated a good fit, while values higher than 0.90 indicated an acceptable adjustment (Schumacker and Lomax, 2012). NNFI greater than or equal to 0.97 indicated a good fit, and 0.95 was an acceptable fit (Jöreskog and Sörbom, 1993). CFI has the same criteria as NNFI (Bollen, 1990; Hu and Bentler, 1999). GFI of 0.95 indicated a good fit, and values greater than 0.90, an acceptable fit (Schumacker and Lomax, 2012). Finally, AGFI 0.90 was indicative of a good fit, and values greater than 0.85 indicated an acceptable fit.

**TABLE 2A:** Indicators of adjustment of victimization and aggression subscales.

Indicator	Estimator Victimization	Estimator Aggression	Correlated	Uncorrelated	Good fit	Acceptable fit
RMSEA	0.03	0.02	0.02	0.10	≤0.05	≤0.08
SRMR	0.05	0.08	0.06	0.23	≤0.1	≤0.1
NFI	0.95	0.94	0.98	0.78	≥0.95	≥0.90
NNFI	0.96	0.95	0.99	0.76	≥0.97	≥0.95
CFI	0.97	0.96	0.99	0.79	≥0.97	≥0.95
GFI	0.99	0.99	1.00	0.96	>0.95	>0.90
AGFI	0.99	0.98	0.99	0.93	>0.90	>0.85

*RMSEA, Root Mean Square Error of Approximation; SRMR, Standardized Root Mean square Residual; NFI, Normed Fit Index; NNFI, Non-normed Fit Index; CFI, Comparative Fit Index; GFI, Goodness of Fit Index; AGFI, Adjusted Goodness of Fit Index.*

**TABLE 2B:** Comparison between the correlated and uncorrelated models of victimization and aggression subscales.

Test	Estimator Victimization	Estimator Aggression	Correlated	Uncorrelated	χ^2^	P value
CHISQ	98.85	92.72	350.6	4801.5	χ^2^(1) = 4450.9	<0.000

*CHISQ, Chi-squared.*

Additionally, the instrument’s reliability was evaluated through the omega coefficient; an acceptable reliability value is 0.65 or more (McDonald, 2013). Also, the average variance extracted was calculated; it was acceptable at a value of at least 0.5 (Fornell and Larcker, 1981).

#### Descriptive Features of the Questionnaire

The items were described by mean, standard deviation, median, skewness and kurtosis coefficients, and the quartiles 1 and 3. These last two parameters are presented in intervals [Q1–Q3], as a robust measure of dispersion. The same analyses were conducted when comparing the psychometric properties of subscales of victimization and perpetration between girls and boys and different socioeconomic statuses.

#### IRT Analysis

The analysis of the psychometric properties of the OBVQ-R was done using IRT for the graded response model (Samejima, 1969) for each of the 10 items included in each subscale. The response alternatives were collapsed into three answer categories: 0 is “never in the last two months at school” (0 = 1), 1 is “it has happened once or twice in the last two months” (1 = 2), and 2 includes the responses “2 or 3 times a month in the last two months,” “about once a week in the last two months” and “several times a week in the last two months” (2 = 3,4,5).

The IRT model estimated three parameters: Alpha, Beta1, and Beta2. Alpha is a discrimination parameter, representing the degree to which the answer categories differentiate between levels of the trait. It remains constant for all the thresholds of the categories of the same item. This discrimination parameter alpha was interpreted according to the following scale: 0 = “no discrimination,” 0.01–0.34 = “very low,” 0.35–0.64 = “low,” 0.65–1.34 = “moderate,” 1.35–1.69 = “high,” and greater than or equal to 1.7 = “very high” (Baker, 2001). On the other hand, Beta1 represents the latent trait needed for the student to pass the threshold from answering 0 (never happened to me in the last two months at school) to 1 (it has happened once or twice in the last two months). In other words, Beta1 refers to the minimum value of the necessary trait to obtain a probability higher than 0.5 in answering option 1. Moreover, Beta2, similar to Beta1, represents the threshold for passing from the answer category of 1 (it has happened once or twice in the last two months) to 2 (it has happened to me 2 or 3 times a month or more in the last two months). With the estimation of these severity parameters, we can order the questions according to their degree of severity; when the threshold is high, the degree of severity will be higher.

#### Differential Item Functioning (DIF) by Gender and SES

We determined if there were items with differential functioning for subgroups. If so, this implies that respondents from different subgroups with the same latent trait level do not have the same probability of responding positively to an item category (Chalmers, 2012). The items can have a different relationship with the principal construct by subgroups (Reise et al., 2005). We evaluated DIF associated with gender and SES independently.

All data analyses were conducted using R 3.5.0. CFA and IRT were performed using lavaan (Rosseel, 2012) and mirt package (Chalmers, 2012), respectively.

#### Concurrent Validity

It was evaluated by analyzing the concordance of seven items of the victimization subscale and seven items of perpetration subscale with similar items in the MIAP. The cyberbullying item of the OBVQ-R was compared to the IEQ, since the MIAP does not include cyberbullying items.

To assess the agreement between each instrument’s questions, we used Cohen’s kappa statistic and its confidence intervals, as well as a hypothesis test in which the null hypothesis proposes that the degree of agreement between the items is random (Landis and Koch, 1977). The degree of agreement between the questions will be interpreted in the following way: if kappa is less than 0, there is “no agreement,” if 0–0.2, “slight agreement,” if 0.2–0.4, “fair agreement,” if 0.4–0.6, “moderate agreement,” if 0.6–0.8, “substantial agreement,” and if 0.8–1.0, “almost perfect agreement” (Landis and Koch, 1977).

## Results

### Dimensionality and Reliability

The OBVQ-R had good parameters of adjustment indicators in both subscales separately (see Table 2A). The unidimensional characteristic of both subscales (victimization and perpetration) was corroborated (see Table 2A). Additionally, the model of the two subscales correlated presented a better fit than the uncorrelated model (see Tables 2A,B, and Figures 1–4).

Finally, the victimization and perpetration subscales have good internal reliability (Cronbach’s alpha = 0.91, ω = 0.81, and Cronbach’s alpha = 0.92, ω = 0.76, respectively), and the average variance extracted was 0.51 and 0.52, respectively.

### Descriptive Features of the Questionnaire

Descriptive statistics of all items of victimization and perpetration subscales are shown in Table 3. All items of victimization and perpetration subscales were mostly comprised of items with asymmetric responses and a high degree of kurtosis. Additionally, results from the factorial analysis showed that the item “I was bullied with mean names, comments, or gestures with a sexual meaning” had the lowest load (0.65) in the victimization subscale, while the lowest load in the perpetration subscale was 0.66, for the item “I called another student(s) mean names, made fun of, or teased him/her in a hurtful way.”

**TABLE 3:** Descriptive Statistics and factor loading.

	Original items							
Questions	*n*	Median	[Q1 – Q3]	Mean	*SD	Kurtosis	Skewness	Factor loading
**Victimization**								
1. I was called mean names, was made fun of, or teased in a hurtful way.	2762	0	[0−1]	0.49	0.99	7.91	2.35	0.75
2. Other students left me out of things on purpose, excluded from their group of friends, or completely ignored me.	2756	0	[0−0]	0.33	0.78	12.43	2.98	0.69
3. I was hit, kicked, pushed, shoved around, or locked indoors.	2751	0	[0−0]	0.13	0.51	32.18	5.08	0.75
4. Other students told lies or spread rumors about me and tried to make others dislike me.	2753	0	[0−1]	0.39	0.84	10.54	2.69	0.72
5. I had money or things taken away from me or damaged.	2755	0	[0−0]	0.18	0.57	21.80	4.07	0.67
6. I was threatened or forced to do things I didn’t want to do.	2755	0	[0−0]	0.15	0.57	28.87	4.85	0.76
7. I was bullied with mean names or comments about my race or color.	2754	0	[0−0]	0.16	0.58	26.95	4.65	0.67
8. I was bullied with mean names, comments, or gestures with a sexual meaning.	2748	0	[0−0]	0.18	0.65	23.55	4.42	0.69
9. I was bullied with cruel messages or hurtful photographs using a cellphone or Internet.	2744	0	[0−0]	0.09	0.40	49.21	6.21	0.65
10. I was bullied in other forms that weren’t mentioned.	2742	0	[0−0]	0.20	0.66	20.80	4.08	0.79
**Aggression**								
1. I called another student(s) mean names, made fun of, or teased him/her in a hurtful way.	2740	0	[0−0]	0.25	0.64	15.95	3.32	0.74
2. I kept him/her out of things on purpose, excluded him or her from my group of friends, or completely ignored him or her.	2737	0	[0−0]	0.17	0.54	25.74	4.34	0.68
3. I hit, kicked, pushed, and shoved him or her around or locked him or her indoors.	2734	0	[0−0]	0.09	0.41	45.64	5.99	0.78
4. I spread false rumors about him/her and tried to make others dislike him/her.	2730	0	[0−0]	0.08	0.38	56.84	6.70	0.74
5. I took money or things from him or her or damaged his/her belongings.	2733	0	[0−0]	0.05	0.28	75.99	7.64	0.67
6. I threatened or forced him/her to do things he/she didn’t want to do.	2730	0	[0−0]	0.03	0.27	129.22	10.37	0.77
7. I bullied him/her with mean names or comments about his/her race or color.	2729	0	[0−0]	0.08	0.38	48.10	6.06	0.67
8. I bullied him/her with mean names, comments, or gestures with sexual meaning.	2726	0	[0−0]	0.04	0.26	71.13	7.43	0.66
9. I bullied him/hers with cruel messages or hurtful photographs using a cellphone or Internet.	2717	0	[0−0]	0.04	0.29	104.07	9.18	0.70
10. I bullied others using other forms that weren’t mentioned.	2707	0	[0−0]	0.07	0.35	60.20	6.74	0.74

*SD, Standard Deviation.

### Item Response Theory analysis

Table 4 shows the descriptive statistics (median and interquartile range) for each question from the victimization and perpetration subscales. Additionally, it presents estimated parameters through IRT (Alpha, Beta1, and Beta2).

**TABLE 4:** Item Response Theory parameter estimates of the OBVQ-R.

Questions	Items in new categorization*		Alpha	Beta1	Beta2
	Median	[Q1 – Q3]			
**Victimization**					
1. I was called mean names, was made fun of, or teased in a hurtful way.	0	[0 – 1]	2.06	0.82	1.65
2. Other students left me out of things on purpose, excluded from their group of friends, or completely ignored me.	0	[0 – 0]	1.8	1.09	2.19
3. I was hit, kicked, pushed, shoved around, or locked indoors.	0	[0 – 0]	2.06	1.8	2.64
4. Other students told lies or spread rumors about me and tried to make others dislike me.	0	[0 – 1]	1.83	0.93	1.99
5. I had money or things taken away from me or damaged.	0	[0 – 0]	1.67	1.7	2.72
6. I was threatened or forced to do things I didn’t want to do.	0	[0 – 0]	2.17	1.71	2.46
7. I was bullied with mean names or comments about my race or color.	0	[0 – 0]	1.62	1.89	2.81
8. I was bullied with mean names, comments, or gestures with a sexual meaning.	0	[0 – 0]	1.86	1.74	2.42
9. I was bullied with cruel messages or hurtful photographs using a cellphone or Internet.	0	[0 – 0]	1.66	2.27	3.23
10. I was bullied in other forms that weren’t mentioned.	0	[0 – 0]	2.36	1.48	2.15
**Aggression**					
1. I called another student(s) mean names, made fun of, or teased him/her in a hurtful way.	0	[0 – 0]	2.19	1.18	2.19
2. I kept him/her out of things on purpose, excluded him or her from my group of friends, or completely ignored him or her.	0	[0 – 0]	1.67	1.66	2.86
3. I hit, kicked, pushed, and shoved him or her around or locked him or her indoors.	0	[0 – 0]	2.41	1.93	2.6
4. I spread false rumors about him/her and tried to make others dislike him/her.	0	[0 – 0]	2.17	2.09	2.9
5. I took money or things from him or her or damaged his/her belongings.	0	[0 – 0]	1.83	2.54	3.44
6. I threatened or forced him/her to do things he/she didn’t want to do.	0	[0 – 0]	2.28	2.54	3.22
7. I bullied him/her with mean names or comments about his/her race or color.	0	[0 – 0]	1.97	2.1	3
8. I bullied him/her with mean names, comments, or gestures with sexual meaning.	0	[0 – 0]	1.87	2.54	3.53
9. I bullied him/hers with cruel messages or hurtful photographs using a cellphone or Internet.	0	[0 – 0]	2.01	2.54	3.37
10. I bullied others using other forms that weren’t mentioned.	0	[0 – 0]	2.15	2.11	3.03

*New categorization: 0, never happened to me in the last two months at school; 1, it has happened once or twice in the last two months; 2, it has happened to me 2 o 3 times a month or more in the last two months. Beta1 represents the latent trait that is needed for the student to pass from 0 to 1; Beta 2 represents the threshold for passing from the answer category of 1 to 2.

The subscale of victimization shows that in eight of the ten items (except items #1 and #4), at least 75% of the students did not suffer bullying at school in the last 2 months. In items #1 and #4, at least 25% of them declared that they were bullied. The 10 items discussed represented the trait of victimization in students. We observed that the discrimination parameters varied between 1.62 (bullying about race and color) and 2.36 (other forms of bullying). Though the 1.62 value was the lowest estimator, it remains high according to Baker’s (2001) classification; therefore, all items in this subscale discriminated the victimization trait very well. In the first (Beta1) and second (Beta 2) thresholds, the item with the lowest latent trait was “#1. I was called mean names, was made fun of, or teased in a hurtful way,” and the item with the highest victimization trait was #9 or cyberbullying.

Every item of the perpetration subscale showed that at least 75% of the students reported not having bullied others at school in the last 2 months. These 10 items had high values or very high values, according to Baker’s (2001) classification, in the trait of perpetration (range between 1.67 and 2.41). Regarding the first threshold (Beta1), the item with the lowest latent trait (1.18) was “#1. I called another student(s) mean names, made fun of, or teased him/her in a hurtful way.” Four items had the highest latent trait (2.54): “#5. I took money or things from him or her or damaged his/her belongings,” “#6. I threatened or forced him/her to do things he/she didn’t want to do,” “#8. I bullied him/her with mean names, comments, or gestures with sexual meaning,” and “#9. I have bullied others with cruel messages or hurtful photographs using a cellphone or Internet.” By analyzing the second threshold (Beta 2), the lowest latent trait (2.19) was “#1. I called another student(s) mean names, made fun of, or teased him/her in a hurtful way,” and “#8. I bullied him/her with mean names, comments, or gestures with sexual meaning” was the highest latent trait (3.53).

### Differential Item Functioning by Gender

Descriptive statistics of all items of victimization and perpetration subscales by gender are shown in Table 5. Independently of gender, all items have asymmetric responses and high degree of kurtosis.

**TABLE 5:** Item parameters accounting for differential item functioning by Gender in subscale of victimization and aggression of the OBVQ-R.

Questions	Gender	*n*	Mean	Median	[Q1-Q3]	*SD	Kurtosis	Skewness	Alpha	Beta1	Beta2
**Victimization**											
1. I was called mean names, was made fun of, or teased in a hurtful way.	Boys	1188	0.56	0	[0 - 1]	1.07	6.48	2.09	2.27	0.73	1.43
	Girl	1577	0.43	0	[0 - 1]	0.92	9.40	2.59	1.90	0.89	1.86
2. Other students left me out of things on purpose, excluded from their group of friends, or completely ignored me.	Boys	1183	0.30	0	[0 - 0]	0.77	13.38	3.18	1.86	1.26	2.09
	Girl	1577	0.36	0	[0 - 0]	0.78	11.85	2.84	1.81	0.96	2.23
3. I was hit, kicked, pushed, shoved around, or locked indoors.	Boys	1182	0.17	0	[0 - 0]	0.58	24.42	4.39	2.15	1.61	2.36
	Girl	1573	0.10	0	[0 - 0]	0.46	42.13	5.84	2.02	1.97	2.93
4. Other students told lies or spread rumors about me and tried to make others dislike me.	Boys	1180	0.33	0	[0 - 0]	0.79	12.15	2.97	1.85	1.14	2.05
	Girl	1577	0.44	0	[0 - 1]	0.87	9.64	2.53	1.95	0.76	1.88
5. I had money or things taken away from me or damaged.	Boys	1181	0.20	0	[0 - 0]	0.61	19.50	3.88	1.73	1.65	2.54
	Girl	1578	0.16	0	[0 - 0]	0.53	23.79	4.21	1.63	1.74	2.89
6. I was threatened or forced to do things I didn’t want to do.	Boys	1182	0.18	0	[0 - 0]	0.60	23.32	4.31	2.19	1.60	2.31
	Girl	1577	0.13	0	[0 - 0]	0.54	34.70	5.37	2.13	1.81	2.62
7. I was bullied with mean names or comments about my race or color.	Boys	1181	0.20	0	[0 - 0]	0.68	20.30	4.08	1.74	1.75	2.47
	Girl	1577	0.13	0	[0 - 0]	0.49	34.91	5.17	1.52	2.03	3.19
8. I was bullied with mean names, comments, or gestures with a sexual meaning.	Boys	1178	0.23	0	[0 - 0]	0.75	17.63	3.82	2.08	1.56	2.08
	Girl	1574	0.15	0	[0 - 0]	0.57	31.08	5.05	1.68	1.92	2.83
9. I was bullied with cruel messages or hurtful photographs using a cellphone or Internet.	Boys	1179	0.09	0	[0 - 0]	0.42	46.27	6.11	1.67	2.25	3.13
	Girl	1569	0.08	0	[0 - 0]	0.38	51.38	6.26	1.66	2.28	3.30
10. I was bullied in other forms that weren’t mentioned.	Boys	1176	0.22	0	[0 - 0]	0.72	17.86	3.81	2.56	1.45	1.96
	Girl	1570	0.18	0	[0 - 0]	0.61	23.61	4.30	2.19	1.53	2.34
**Aggression**											
1. I called another student(s) mean names, made fun of, or teased him/her in a hurtful way.	Boys	1177	0.33	0	[0 - 0]	0.71	12.58	2.87	1.90	1.02	2.16
	Girl	1567	0.20	0	[0 - 0]	0.58	19.82	3.77	2.52	1.31	2.22
2. I kept him/her out of things on purpose, excluded him or her from my group of friends, or completely ignored him or her.	Boys	1174	0.16	0	[0 - 0]	0.53	27.77	4.54	2.11	1.58	2.55
	Girl	1567	0.18	0	[0 - 0]	0.55	24.47	4.21	1.51	1.69	3.06
3. I hit, kicked, pushed, and shoved him or her around or locked him or her indoors.	Boys	1173	0.12	0	[0 - 0]	0.48	33.50	5.09	2.48	1.72	2.38
	Girl	1565	0.06	0	[0 - 0]	0.34	61.92	7.02	2.24	2.17	2.89
4. I spread false rumors about him/her and tried to make others dislike him/her.	Boys	1171	0.07	0	[0 - 0]	0.36	54.93	6.51	2.54	1.97	2.75
	Girl	1563	0.08	0	[0 - 0]	0.39	57.47	6.80	1.98	2.18	3.02
5. I took money or things from him or her or damaged his/her belongings.	Boys	1171	0.06	0	[0 - 0]	0.31	52.40	6.40	1.91	2.35	3.12
	Girl	1566	0.04	0	[0 - 0]	0.25	108.72	9.08	1.73	2.74	3.82
6. I threatened or forced him/her to do things he/she didn’t want to do.	Boys	1170	0.04	0	[0 - 0]	0.29	108.35	9.40	2.11	2.49	3.22
	Girl	1564	0.03	0	[0 - 0]	0.25	151.21	11.32	2.46	2.58	3.22
7. I bullied him/her with mean names or comments about his/her race or color.	Boys	1169	0.12	0	[0 - 0]	0.45	35.95	5.19	1.62	2.04	3.19
	Girl	1564	0.06	0	[0 - 0]	0.32	64.16	7.06	2.38	2.17	2.88
8. I bullied him/her with mean names, comments, or gestures with sexual meaning.	Boys	1168	0.07	0	[0 - 0]	0.34	46.52	5.98	1.65	2.38	3.43
	Girl	1562	0.02	0	[0 - 0]	0.18	101.41	9.07	2.23	2.68	3.67
9. I bullied him/hers with cruel messages or hurtful photographs using a cellphone or Internet.	Boys	1164	0.05	0	[0 - 0]	0.33	88.08	8.63	2.57	2.28	2.91
	Girl	1557	0.04	0	[0 - 0]	0.25	112.13	9.25	1.73	2.76	3.79
10. I bullied others using other forms that weren’t mentioned.	Boys	1165	0.08	0	[0 - 0]	0.39	56.16	6.62	2.17	2.10	2.92
	Girl	1546	0.07	0	[0 - 0]	0.32	61.38	6.70	2.27	2.07	3.03

*SD, Standard Deviation.

The victimization subscale items have a discrimination parameter estimate between 1.52 and 2.19 for girls and between 1.67 and 2.56 for boys. In general, the discrimination parameters of all items (except “#4. Other students told lies or spread rumors about me and tried to make others dislike me”) were higher among boys than girls. We also found that the item “#8. I was bullied with mean names, comments, or gestures with a sexual meaning” had the highest difference between girls and boys, followed by the item “#1. I was called mean names, was made fun of, or teased in a hurtful way.” In general, most items showed that the parameters Beta 1 and Beta 2 were higher in girls than in boys (see Table 6).

**TABLE 6:** Item parameters accounting for differential item functioning by ses in subscale of victimization and aggression of the OBVQ-R.

Questions	SES	*n*	Mean	Median	[Q1-Q3]	*SD	Kurtosis	Skewness	Alpha	Beta1	Beta2
**Victimization**											
1. I was called mean names, was made fun of, or teased in a hurtful way.	Low	767	0.80	0	[0 – 1]	1.27	4.15	1.56	2.05	0.41	1.17
	Medium	966	0.40	0	[0 – 0]	0.88	10.18	2.69	2.05	0.91	1.83
	High	1029	0.33	0	[0 – 0]	0.77	11.74	2.89	1.98	1.08	2.01
2. Other students left me out of things on purpose, excluded from their group of friends, or completely ignored me.	Low	763	0.41	0	[0 – 0]	0.93	9.29	2.61	2.11	0.97	1.72
	Medium	961	0.30	0	[0 – 0]	0.70	14.26	3.13	1.45	1.23	2.76
	High	1032	0.30	0	[0 – 0]	0.71	13.38	3.04	1.94	1.08	2.20
3. I was hit, kicked, pushed, shoved around, or locked indoors.	Low	759	0.24	0	[0 – 0]	0.74	16.26	3.62	2.07	1.44	2.13
	Medium	961	0.11	0	[0 – 0]	0.43	36.39	5.22	1.66	2.02	3.20
	High	1031	0.07	0	[0 – 0]	0.34	60.15	6.77	2.42	2.00	2.92
4. Other students told lies or spread rumors about me and tried to make others dislike me.	Low	765	0.52	0	[0 – 1]	1.02	7.10	2.20	2.05	0.79	1.52
	Medium	961	0.40	0	[0 – 1]	0.80	10.72	2.64	1.71	0.86	2.13
	High	1027	0.30	0	[0 – 0]	0.70	14.86	3.20	1.71	1.14	2.43
5. I had money or things taken away from me or damaged.	Low	765	0.24	0	[0 – 0]	0.68	14.93	3.38	2.10	1.40	2.14
	Medium	960	0.16	0	[0 – 0]	0.54	26.34	4.49	1.62	1.82	2.86
	High	1030	0.15	0	[0 – 0]	0.50	25.70	4.34	1.38	1.95	3.37
6. I was threatened or forced to do things I didn’t want to do.	Low	763	0.23	0	[0 – 0]	0.75	18.07	3.88	2.54	1.45	2.00
	Medium	960	0.13	0	[0 – 0]	0.51	33.57	5.18	2.16	1.78	2.60
	High	1032	0.11	0	[0 – 0]	0.45	37.33	5.36	1.88	1.92	2.88
7. I was bullied with mean names or comments about my race or color.	Low	764	0.28	0	[0 – 0]	0.80	14.17	3.34	1.74	1.44	2.15
	Medium	961	0.14	0	[0 – 0]	0.54	31.58	5.04	1.39	2.18	3.30
	High	1029	0.09	0	[0 – 0]	0.38	47.59	5.93	1.55	2.24	3.56
8. I was bullied with mean names, comments, or gestures with a sexual meaning.	Low	760	0.22	0	[0 – 0]	0.75	18.73	3.98	1.51	1.84	2.51
	Medium	958	0.18	0	[0 – 0]	0.63	24.72	4.50	2.34	1.56	2.29
	High	1030	0.16	0	[0 – 0]	0.60	27.05	4.70	2.02	1.76	2.40
9. I was bullied with cruel messages or hurtful photographs using a cellphone or Internet.	Low	757	0.12	0	[0 – 0]	0.51	36.70	5.52	1.99	1.97	2.65
	Medium	958	0.08	0	[0 – 0]	0.35	51.26	6.16	1.44	2.46	3.73
	High	1029	0.07	0	[0 – 0]	0.34	54.02	6.41	1.61	2.40	3.41
10. I was bullied in other forms that weren’t mentioned.	Low	757	0.28	0	[0 – 0]	0.82	14.16	3.38	2.53	1.33	1.82
	Medium	957	0.16	0	[0 – 0]	0.55	26.00	4.47	2.47	1.53	2.29
	High	1028	0.18	0	[0 – 0]	0.62	23.40	4.33	2.27	1.55	2.30
**Aggression**											
1. I called another student(s) mean names, made fun of, or teased him/her in a hurtful way	Low	759	0.45	0	[0 – 1]	0.85	9.06	2.39	2.22	0.70	1.69
	Medium	953	0.20	0	[0 – 0]	0.57	19.88	3.73	2.13	1.35	2.43
	High	1028	0.16	0	[0 – 0]	0.48	22.12	3.90	1.81	1.60	2.86
2. I kept him/her out of things on purpose, excluded him or her from my group of friends, or completely ignored him or her.	Low	757	0.22	0	[0 – 0]	0.66	18.98	3.83	2.06	1.44	2.22
	Medium	951	0.17	0	[0 – 0]	0.56	28.31	4.62	1.62	1.73	2.95
	High	1029	0.14	0	[0 – 0]	0.42	21.40	3.74	1.56	1.75	3.49
3. I hit, kicked, pushed, and shoved him or her around or locked him or her indoors.	Low	755	0.18	0	[0 – 0]	0.58	19.77	3.86	2.39	1.49	2.08
	Medium	951	0.06	0	[0 – 0]	0.35	74.81	7.74	2.39	2.16	2.85
	High	1028	0.04	0	[0 – 0]	0.26	94.66	8.46	2.00	2.44	3.61
4. I spread false rumors about him/her and tried to make others dislike him/her.	Low	753	0.13	0	[0 – 0]	0.53	32.23	5.11	2.88	1.65	2.19
	Medium	948	0.06	0	[0 – 0]	0.31	59.39	6.76	2.06	2.24	3.14
	High	1029	0.05	0	[0 – 0]	0.29	91.43	8.28	1.37	2.92	4.56
5. I took money or things from him or her or damaged his/her belongings.	Low	754	0.06	0	[0 – 0]	0.34	65.23	7.19	1.95	2.32	3.03
	Medium	951	0.04	0	[0 – 0]	0.25	106.38	9.20	1.59	3.00	3.82
	High	1028	0.05	0	[0 – 0]	0.25	45.80	6.06	2.48	2.16	3.16
6. I threatened or forced him/her to do things he/she didn’t want to do.	Low	753	0.04	0	[0 – 0]	0.33	96.12	9.20	3.18	2.25	2.57
	Medium	949	0.04	0	[0 – 0]	0.30	117.47	0.06	3.35	2.27	2.70
	High	1028	0.03	0	[0 – 0]	0.17	103.67	8.70	1.58	3.07	5.25
7. I bullied him/her with mean names or comments about his/her race or color.	Low	752	0.16	0	[0 – 0]	0.50	23.43	4.07	2.21	1.51	2.51
	Medium	950	0.07	0	[0 – 0]	0.34	64.54	6.93	1.46	2.55	3.76
	High	1027	0.04	0	[0 – 0]	0.30	93.62	8.91	1.82	2.83	3.36
8. I bullied him/her with mean names, comments, or gestures with sexual meaning.	Low	751	0.07	0	[0 – 0]	0.31	44.14	5.89	2.12	2.15	3.18
	Medium	949	0.03	0	[0 – 0]	0.20	54.89	6.89	1.71	2.83	3.97
	High	1026	0.04	0	[0 – 0]	0.27	91.35	8.55	1.91	2.60	3.39
9. I bullied him/hers with cruel messages or hurtful photographs using a cellphone or Internet.	Low	746	0.06	0	[0 – 0]	0.35	70.39	7.73	2.76	2.13	2.60
	Medium	945	0.04	0	[0 – 0]	0.31	122.94	10.18	1.80	2.70	3.72
	High	1026	0.03	0	[0 – 0]	0.21	73.52	7.72	1.71	2.85	4.07
10. I bullied others using other forms that weren’t mentioned.	Low	747	0.13	0	[0 – 0]	0.48	34.71	5.12	2.17	1.74	2.60
	Medium	939	0.05	0	[0 – 0]	0.31	74.91	7.56	2.15	2.30	3.08
	High	1021	0.05	0	[0 – 0]	0.26	84.56	7.80	1.92	2.43	3.81

*SD, Standard Deviation.

Regarding the perpetration subscale, we found that the cyberbullying item (#9) had the highest difference between girls (1.73) and boys (2.57), followed by item “#7. I was bullied with mean names or comments about my race or color” (girls, 2.38 and boys, 1.62). Most items showed that the parameters Beta 1 and Beta 2 were higher in girls than in boys (see Table 5).

### Differential Item Functioning by SES

Descriptive statistics of all items of the victimization and perpetration subscales by SES are shown in Table 6. All items have asymmetric responses and a high degree of kurtosis, especially among students coming from middle- and high-income schools.

The victimization subscale items had discrimination parameter estimates between 1.55 and 2.54 for students of low-income schools, between 1.39 and 2.53 for students of middle-income schools, and between 1.38 and 2.42 for students of high-income schools. Additionally, most discrimination parameter estimates were higher among students coming from low-income schools, except item “#3. I was hit, kicked, pushed, shoved around, or locked indoors,” which was higher in high-income schools; and item “#8. I was bullied with mean names, comments, or gestures with a sexual meaning,” which was higher in middle-income schools. Most items showed that the parameters Beta 1 and Beta 2 were higher in students coming from middle- or high-income schools than in those students coming from low-income schools (see Table 6), except item “#8. I was bullied with mean names, comments, or gestures with a sexual meaning,” which was higher among students attending low-income schools.

Regarding the perpetration subscale, the items had discrimination parameter estimates between 1.95 and 2.88 for students of low-income schools, between 1.46 and 3.35 for students of middle-income schools, and between 1.37 and 2.48 for students of high-income schools. Most discrimination parameter estimates were higher among students coming from low-income schools, except “#5. I took money or things from him or her or damaged his/her belongings, which was higher in high-income schools” and “#6. I threatened or forced him/her to do things he/she didn’t want to do,” which was higher in middle-income schools. All items showed that the parameters Beta 1 and Beta 2 were higher in students coming from middle- or high-income schools than in those students coming from low-income schools (see Table 6).

### Concurrent Evidence of Validation

In the victimization subscale, there was a concordance from 0.14 to 0.36 for similar items; all the concordances are statistically significant (*p*-values < 0.001). Five out of eight items have a “fair agreement.” On the other hand, four of the eight items analyzed in the perpetration subscale have a “fair agreement” with a range concordance between 0.22 and 0.32 and two items (“I threatened or forced him/her to do things he/she didn’t want to do” and “I bullied him/her with mean names, comments, or gestures with sexual meaning”) had no association between instruments. See Table 7.

**TABLE 7:** Concurrent analysis between OBVQ-R and School Violence between Peers Questionnaire (MIAP)/Internet Experiences Questionnaire (IEQ).

OBVQ-R	MIAP/IEQ	*n*	Kappa*	CI	*p*-value
**Victimization**					
I was called mean names, was made fun of, or teased in a hurtful way.	[MIAP] They insult me or put me nicknames that offend or ridicule me.	2,398	0.36	(0.32 – 0.40)	0.000
Other students left me out of things on purpose, excluded from their group of friends, or completely ignored me.	[MIAP] They ignore me (“ice law”) or don’t let me participate.	2,391	0.29	(0.24 – 0.33)	0.000
I was hit, kicked, pushed, shoved around, or locked indoors.	[MIAP] They hit me.	2,386	0.29	(0.22 – 0.36)	0.000
Other students told lies or spread rumors about me and tried to make others dislike me.	[MIAP] They speak ill of me.	2,385	0.28	(0.24 – 0.32)	0.000
I had money or things taken away from me or damaged.	[MIAP] They hide things, break things, or rob me.	2,775	0.18	(0.13- 0.23)	0.000
I was threatened or forced to do things I didn’t want to do.	[MIAP] They threat me just to get me scared, they force me to do things I do not want to do with threats (bring money, do their homework, ask my sneakers, etc.), they force me to do things (miss classes, get out of class).	2,389	0.18	(0.08 – 0.27)	0.000
I was bullied with mean names, comments, or gestures with a sexual meaning.	[MIAP] They sexually harass me.	2,375	0.14	(0.04 – 0.24)	0.000
I have been bullied with cruel messages or hurtful photographs using a cellphone or Internet.	[IEQ] I have been a victim of bullying through text messages (using cellphone or WhatsApp), or internet (posting on a website or blog) or sending pictures or videos without your permission (using cellphone).	2,247	0.34	(0.25 – 0.42)	0.000
**Aggression**					
I called another student(s) mean names, made fun of, or teased him/her in a hurtful way.	[MIAP] I insult or put nicknames that offend or ridicule him/her.	2,373	0.32	(0.27 – 0.37)	0.000
I kept him/her out of things on purpose, excluded him or her from my group of friends, or completely ignored him or her.	[MIAP] I ignore (“ice law”) or do not let participate him/her.	2,375	0.22	(0.17 – 0.27)	0.000
I hit, kicked, pushed, and shoved him or her around or locked him or her indoors.	[MIAP] I hit him/her.	2,372	0.26	(0.17 – 0.36)	0.000
I spread false rumors about him/her and tried to make others dislike him/her.	[MIAP] I speak bad about him or her.	2,368	0.13	(0.07 – 0.20)	0.000
I took money or things from him or her or damaged his/her belongings.	[MIAP] I hide, break or steal things from him/her.	2,368	0.13	(0.04 - 0.22)	0.003
I threatened or forced him/her to do things he/she didn’t want to do.	[MIAP] I threat him/her just to make /him/her afraid, I force him/her to things with threats (ask money, ask to do homework, ask for their sneakers, etc.), I force him/her to do things (not to go to class, get out from class).	2,368	0.09	(-0.11 – 0.28)	0.201
I bullied him/her with mean names, comments, or gestures with sexual meaning.	[MIAP] I sexually harass him/her.	2,363	0.09	(-0.10 – 0.27)	0.183
I have bullied others with cruel messages or hurtful photographs using a cellphone or Internet	[IEQ] I have bullied others through text messages (using cellphone or WhatsApp), or internet (posting on a website or blog) or sending pictures or videos without your permission (using cellphone).	2,272	0.22	(0.09 – 0.35)	0.001

*Kappa interpretation: 0, “there is no agreement”; 0-0.2, “slight agreement”; 0.2-0.4, “fair agreement”; 0.4- 0.6, “moderate agreement”; 0.6-0.8, “substantial agreement”; 0.8-1.0, “almost perfect agreement.” CI, Confidence Interval.

The item about racist bullying was not included in these analyses because the MIAP does not ask about that form of bullying.

## Discussion

This is the first study that explores the validity and reliability of the OBVQ-R in Chile. This study confirms the two-factor structure and unidimensionality of the victimization and perpetration subscales (Solberg and Olweus, 2003; Breivik and Olweus, 2015). All items should be considered as key elements of each subscale. Additionally, the model considering both subscales correlated had a better fit than the model exploring both subscales uncorrelated. We also found differences by gender and socioeconomic status of the schools in the expression of the victimization and perpetration traits. The concurrent validation conducted in our study found that the Cohen’s kappa statistic score was in the range of slight or higher agreement between compared instruments. The reliability of the instrument seems good for both subscales.

All items of the questionnaire are important for each subscale, having a high factor loading and high or very high discrimination parameter estimates. In the victimization subscale, the items “I was called mean names,” “I was hit, kicked and pushed,” and “I was threatened or forced to do things” were those with the highest discrimination estimates. It is worth mentioning that the item “other forms of bullying” had the highest alpha score. Some other authors have removed this item from the analyses (Breivik and Olweus, 2015), but our results support the idea of keeping this item as part of the subscale but include additional questions to understand better what students feel about this item. On the other hand, in the perpetration subscale, the item “I was threatened or forced” had the highest alpha score. Several studies have found different discrimination items (Breivik and Olweus, 2015; Resett et al., 2015), highlighting the importance of performing validation studies in different countries to take into account cultural differences. On the other hand, in both subscales, the IRT analysis showed that the item with the lowest-severity parameter was “I was called mean names” and “I called another student(s) mean names,” which may be explained because verbal aggression is one of the most common forms of bullying. In addition, the items with the highest-severity parameters in both subscales were different. In the victimization subscale, the highest-severity parameter was cyberbullying, but in the perpetration subscale it was sexual bullying. The fact that being a victim of cyberbullying was considered a severe form of bullying may explain the findings of other authors about the relationship between cyberbullying and suicidal ideation and attempts (John et al., 2018; Peng et al., 2019). Regarding the perpetration subscale, bullying others with “mean names, comments, or gestures with sexual meaning” can be considered a final step in the aggression possibilities and a more severe behavioral pattern of the perpetrators. These results are similar to those reported by Breivik and Olweus (2015).

We were able to compare different models of the structure of the questionnaire, finding that the best model corresponds to two correlated dimensions of bullying, victimization, and perpetration. This structure has been found in other studies (Kyriakides et al., 2006; Breivik and Olweus, 2015). Additionally, we found that both subscales were correlated, which may be explained because many students who considered themselves as victims were also perpetrators.

We found that boys responded with a lower-severity parameter in almost every item. In the victimization subscale, the exception was the rumors item, in which girls showed a lower-severity parameter than boys. In the perpetration subscale, in the item about threats or being forced to do things, boys and girls had the same-severity parameter, and in the item about racial bullying, girls had a lower-severity parameter than boys. The latter may be explained because boys are more involved in bullying than girls, which is supported by other studies (Zych et al., 2015). About the rumors item, we did not expect to find differences between subscales (in the victimization subscale, girls had lower severity, and in the perpetration subscale, they had higher severity than in boys). Previous literature shows that girls are more involved in relational forms of bullying, either as victims or bullies (Wang et al., 2009). An explanation of this may be that female students in Chile are less likely to recognize themselves as spreading rumors about others because they considered these actions culturally unacceptable, similar to what happens with physical bullying among girls. However, they did recognize being the target of rumors.

We found differences in the expression of bullying by SES. Generally, different forms of victimization and perpetration were more common among students coming from low-income schools. Students from low-income families may have been exposed to a higher proportion of family conflicts than students from families with more economic resources. For instance, there is evidence that children and adolescents of low SES families had a higher chance of being exposed to domestic violence (Cunradi et al., 2002) and harsher punishment (Straus and Stewart, 1999), which may shape how they interact with others in their school context. Moreover, students from high-income families may have a better development of problem-solving skills and prosocial norms and values (Galobardes et al., 2006a,b). It is important to have local information, because other countries do not have the SES differences in the bullying experience that we found in Chile, and this instrument would allow us to conduct future comparisons (Tippett and Wolke, 2014).

In terms of the concurrent analysis of the OBVQ-R with the MIAP and the IEQ, we found differences between both subscales. The concordance between the items of the three instruments for the victimization subscale ranged from “slight agreement” to “fair agreement” but was significant in all cases. We could say that students who were perceived as victims of bullying were also perceived as victims of school violence in general; however, it seems that both concepts are not quite the same for them considering the small degree of agreement. These results highlight the idea that school violence and bullying are perceived as two different concepts by adolescents. In the perpetration subscale, the concordance between the items of three instruments was between 0.09 and 0.32. Only in two items, there was no correlation (#6. I threatened or forced him/her to do things he/she didn’t want to do, and #8. I bullied him/her with mean names, comments, or gestures with sexual meaning”). Regarding these last two items, the formulation of the sentences was slightly different between the OBVQ-R and the MIAP. For instance, when it comes to threats, in the MIAP, both items refer to actions one can do to force another person to do things, including a range of examples. The OBVQ-R takes a more general approach, without specifying the action or the consequence of the behavior. For the items about sexual bullying, the MIAP asks for “sexual harassment,” unlike the OBVQ-R, which asks for “being bullied with names, comments or gestures with sexual meaning.” Other authors also take the view that the definition of sexual harassment is broad and it includes a range of behaviors (McMaster et al., 2002; Chiodo et al., 2009). These researchers also postulate that sexual harassment may include in the same definition severe (e.g., sexual assault) and less severe behaviors (e.g., sexual jokes or comments) (McMaster et al., 2002). In another study, Shute et al. (2008) asked adolescents about sexual harassment and victimization and found that physical sexual harassment was not as frequent as verbal sexual harassment. According to these studies, sexual harassment is a concept that may include many and varied behaviors; therefore, in our study, students may have considered a more general definition of sexual harassment (in the MIAP), taking together severe and less severe actions, while in the OBVQ-R, students may have answered it according to a more specific and narrower concept.

We can mention several limitations of this study. First, we used retrospective, self-reported measures in an adolescent population, which may introduce reporting bias (Pokorski et al., 1994) and social desirability bias (Brittingham et al., 1998), especially for the aggressive behaviors. However, the biases mentioned above do not threaten the validity of self-reported measurements among students (Brener et al., 2003). Additionally, when administering the OBVQ-R scale, research assistants did not report any complaints about the comprehension of items. Second, this study followed a cross-sectional design, which does not allow inferences about the long-term effects of these behaviors. Third, a 28% (9/32) of the invited schools agreed to participate. This may have introduced a selection bias in the results. However, we managed to include schools from different socioeconomic backgrounds and with similar participation proportion in the total sample of students, reducing the risk of bias. Fourth, the MIAP and IEQ questionnaires, used to compare the information gathered with the OBVQ-R, were the only available instruments with results published in scientific journals in Chile. Even though we recognize that the psychometric properties and features of these instruments were not ideal, we considered it important to make available to the audience and potential users the comparison between the instruments to provide as much information as possible to make informed decisions in the future when selecting a questionnaire to evaluate bullying experiences. Fifth, even though the sample size was large and aimed to represent the adolescent population in Chile, there could be regional differences in bullying among adolescents living in the North and South regions in Chile. Finally, it is important to mention that there is one item that requires further exploration for future research: #10, exploring “other” forms of bullying. Students did not have any problem answering this item; however, it is difficult to know, as it is stated in the questionnaire, what the other forms of bullying the students are referring to. Therefore, we suggest continuing using this item but including a new question where students can write the other forms of bullying they are referring to, to understand this item better.

## Conclusion

The OBVQ-R appears to have a good item structure, validity, and reliability when assessing bullying among adolescent students in Chile. We have provided evidence that this is a two-factor structure questionnaire, and the victimization and perpetration subscales gather information about several forms of bullying. This instrument may be useful for studying the prevalence of bullying and assessing the effectiveness of anti-bullying programs.

## Data Availability Statement

The raw data supporting the conclusions of this article will be made available by the authors, without undue reservation.

## Ethics Statement

The studies involving human participants were reviewed and approved by The Ethical Committee of the University de los Andes, Chile. Written informed consent to participate in this study was provided by the participants’ legal guardian/next of kin.

## Author Contributions

JG, CS, CR-B, and RA conceived and designed the study and supervised data collection. JG, MG, and DV analyzed and interpreted the data and produced the draft of the manuscript. RA supervised all steps in the study. All authors provided a critical revision of the manuscript.

## Conflict of Interest

The authors declare that the research was conducted in the absence of any commercial or financial relationships that could be construed as a potential conflict of interest.
