# Supplementary material for: Validation of the Revised Olweus Bully/Victim Questionnaire (OBVQ-R) Among Adolescents in Chile
Source: Front Psychol. 2021 Apr 12;12:578661. doi: 10.3389/fpsyg.2021.578661 (PMC8072054; doi:10.3389/fpsyg.2021.578661)
Supplement: Supplementary file 1 [file Data_Sheet_1.PDF]

ID:

Cuestionario Escolares

Proyecto AKA-EDU/15

Uso interno
Nombre del evaluador:

Fecha de hoy	Día:	Mes:	Año:
Nombre completo:			
RUT:			
Edad:			
Fecha de nacimiento	Día:	Mes:	Año:

Curso: ◯ 3 ◯ 4 ◯ 5 ◯ 6 ◯ 7 ◯ 8

Letra del curso: ◯ A ◯ B ◯ C ◯ D ◯ E

Instrucciones generales

Queremos conocer tus opiniones y preocupaciones en relación a tu experiencia en el colegio. Es muy importante que respondas este cuestionario con sinceridad, y que intentes responder todas las preguntas. Si tienes preguntas, levanta la mano.

No existen respuestas buenas o malas, ya que no es un examen.

A. A continuación, encontrarás preguntas sobre tu vida en la escuela. Debajo de cada pregunta hay varias respuestas. Contesta a la pregunta rellenando el círculo junto a la respuesta que mejor describa lo que piensas o sientes. Por ejemplo, en la pregunta 1, si en verdad te desagrada la escuela, rellena el círculo que corresponda a “Me desagrada mucho”. Si en verdad te gusta la escuela, rellena el círculo que corresponda a “Me agrada mucho”, etc. **Sólo rellena un círculo por cada pregunta.** Procura rellenar el círculo sin salirte de la raya.

Ahora, rellena el círculo junto a la respuesta que mejor describa cómo te sientes respecto de la escuela.

1. ¿Qué te parece la escuela?
   - ○ Me desagrada mucho
   - ○ No me agrada
   - ○ Ni me agrada ni me desagrada
   - ○ Me agrada
   - ○ Me agrada mucho

Si **rellenas el círculo equivocado**, puedes cambiar tu respuesta así: Pon una “X” que atraviese el círculo así ⊗ y después rellena el círculo que esté junto a la respuesta correcta.

A veces será difícil decidir cuál es la mejor respuesta, pero trata de contestarla lo mejor que puedas.

La mayoría de las preguntas tienen que ver con **tu vida en la escuela en los últimos dos meses.** Cuando contestes las preguntas piensa cómo ha sido tu vida en la escuela durante los últimos dos meses y **no únicamente cómo es en el presente**.

2. ¿Eres niño o niña?
   - ○ Niña
   - ○ Niño

3. ¿Cuántos buenos amigos o amigas tienes en tu clase o clases?
   - ○ Ninguno/a
   - ○ Tengo un buen amigo o amiga
   - ○ Tengo dos o tres buenos amigos o amigas
   - ○ Tengo cuatro o cinco buenos amigos o amigas
   - ○ Tengo seis o más buenos amigos o amigas

¿Cuándo un estudiante sufre bullying por otros estudiantes?

Después de este cuadro, verás algunas preguntas sobre sufrir bullying. Primero, veamos lo que se entiende por bullying. Recuerda esta explicación sobre el bullying cuando respondas las preguntas.

Un estudiante está sufriendo bullying cuando uno o más alumnos:

- dicen cosas malintencionadas o hirientes sobre él/ella, se ríen o se burlan de él/ella, o le ponen apodos o sobrenombres ofensivos
- le ignoran por completo, le excluyen de su grupo de amigos, o le dejan fuera a propósito
- le dan golpes, patadas, empujones, o le mandonean o, por ejemplo, le encierran en una habitación
- tratan de crearle mala fama ante los demás alumnos diciendo mentiras sobre él/ella o enviándole notas crueles
- le hacen otras cosas hirientes distintas a las mencionadas anteriormente

Se trata de bullying cuando esto se produce de manera **repetida y a la víctima le cuesta defenderse**. También es bullying cuando se molesta en repetidas ocasiones a un alumno de manera malintencionada e hiriente.

¿Qué no es bullying? Molestar a alguien de manera amigable y en broma no es bullying. Tampoco se considera bullying cuando estudiantes con una fuerza más o menos parecida discuten o pelean por voluntad propia.

4. ¿Qué tan frecuentemente has sufrido bullying en la escuela **en los últimos dos meses**?
   - ○ No he sufrido bullying en la escuela en los últimos dos meses
   - ○ Sólo ha ocurrido una o dos veces
   - ○ 2 o 3 veces al mes
   - ○ Como una vez a la semana
   - ○ Varias veces a la semana

¿Has sufrido bullying en la escuela en los últimos dos meses en alguna de las siguientes maneras?
(preguntas 5-13)

Recuerda que el bullying es cuando un estudiante hacer sentir mal a otro a propósito y repetidamente.

5. Me pusieron apodos ofensivos, me ridiculizaron o me molestaron de forma hiriente.
   - ○ No me ha ocurrido en los últimos dos meses
   - ○ Sólo ha ocurrido una o dos veces
   - ○ 2 o 3 veces al mes
   - ○ Como una vez a la semana
   - ○ Varias veces a la semana

6. Otros estudiantes me excluyeron a propósito de lo que estaban haciendo o de su grupo de amigos, o me ignoraron por completo.
   - ○ No me ha ocurrido en los últimos dos meses
   - ○ Sólo ha ocurrido una o dos veces
   - ○ 2 o 3 veces al mes
   - ○ Como una vez a la semana
   - ○ Varias veces a la semana

7. Me pegaron, patearon, empujaron o encerraron dentro de la sala de clases.
   - ○ No me ha ocurrido en los últimos dos meses
   - ○ Sólo ha ocurrido una o dos veces
   - ○ 2 o 3 veces al mes
   - ○ Como una vez a la semana
   - ○ Varias veces a la semana

8. Otros estudiantes dijeron mentiras o esparcieron rumores falsos acerca de mí y trataron que les cayera mal a otros.
   - ○ No me ha ocurrido en los últimos dos meses
   - ○ Sólo ha ocurrido una o dos veces
   - ○ 2 o 3 veces al mes
   - ○ Como una vez a la semana
   - ○ Varias veces a la semana

9. Me quitaron mi dinero u otras cosas o dañaron mis pertenencias.
   - ○ No me ha ocurrido en los últimos dos meses
   - ○ Sólo ha ocurrido una o dos veces
   - ○ 2 o 3 veces al mes
   - ○ Como una vez a la semana
   - ○ Varias veces a la semana

10. Me amenazaron o me forzaron a hacer cosas que no quería hacer.
   - ○ No me ha ocurrido en los últimos dos meses
   - ○ Sólo ha ocurrido una o dos veces
   - ○ 2 o 3 veces al mes
   - ○ Como una vez a la semana
   - ○ Varias veces a la semana

11. Me insultaron con comentarios acerca de mi país de origen, raza o color de piel.
   - ○ No me ha ocurrido en los últimos dos meses
   - ○ Sólo ha ocurrido una o dos veces
   - ○ 2 o 3 veces al mes
   - ○ Como una vez a la semana
   - ○ Varias veces a la semana

12. Me insultaron con comentarios o gestos con tonos sexuales.
   - ○ No me ha ocurrido en los últimos dos meses
   - ○ Sólo ha ocurrido una o dos veces
   - ○ 2 o 3 veces al mes
   - ○ Como una vez a la semana
   - ○ Varias veces a la semana

12a. Me han hecho bullying mandándome mensajes o fotos crueles o hirientes por medio de un celular, teléfono o Internet (computadora). (Por favor, recuerda que no es bullying cuando esto se hace bromeando y de un modo amistoso).

- ○ No me ha ocurrido en los últimos dos meses
- ○ Sólo ha ocurrido una o dos veces
- ○ 2 o 3 veces al mes
- ○ Como una vez a la semana
- ○ Varias veces a la semana

12b. Si te han hecho bullying por medio de Internet o un celular, ¿cómo fue?

- ○ Sólo por un celular
- ○ Sólo por medio del Internet
- ○ De las dos maneras

13. Me han hecho bullying de otra forma (alguna que no haya sido mencionada anteriormente).

- ○ No me ha ocurrido en los últimos dos meses
- ○ Sólo ha ocurrido una o dos veces
- ○ 2 o 3 veces al mes
- ○ Como una vez a la semana
- ○ Varias veces a la semana

14. ¿**En qué clase o clases** está el estudiante o estudiantes que te hacen bullying?

- ○ No he sufrido bullying en la escuela en los últimos dos meses
- ○ En mi clase
- ○ En una clase diferente, pero en el mismo grado (año) escolar
- ○ En un grado/s más alto
- ○ En un grado/s más bajo
- ○ En diferentes grados

15. ¿Has sufrido bullying por parte de **niños o niñas**?

- ○ No me han hecho bullying en la escuela en los últimos dos meses
- ○ Principalmente una niña
- ○ Varias niñas
- ○ Principalmente un niño
- ○ Varios niños
- ○ Niños y niñas por igual

16. Por lo general, ¿cuántos estudiantes te han hecho bullying?

- ○ No he sufrido bullying en la escuela en los últimos dos meses
- ○ Principalmente un estudiante
- ○ Un grupo de 2-3 estudiantes
- ○ Un grupo de 4-9 estudiantes
- ○ Un grupo de 10 o más estudiantes
- ○ Varios estudiantes o grupos de estudiantes distintos

17. ¿**Cuánto tiempo** has estado sufriendo de bullying?
  - ○ No he sufrido bullying en la escuela en los últimos dos meses
  - ○ Una o dos semanas
  - ○ Un mes aproximadamente
  - ○ Aproximadamente 6 meses
  - ○ Aproximadamente un año
  - ○ Ha estado sucediendo por varios años

18. ¿En **qué lugar** has sufrido bullying?
  - ○ No he sufrido de bullying en la escuela en los últimos dos meses
  - ○ He sufrido de bullying **en uno o más de los siguientes lugares** en los últimos dos meses

  Por favor, rellena los círculos de todos los lugares donde te han hecho bullying:

  - ○ 18a. En el área de juegos/en el patio (en el recreo o en los ratos de descanso)
  - ○ 18b. En los pasillos
  - ○ 18c. En clase (cuando el profesor/a estaba presente)
  - ○ 18d. En clase (cuando el profesor/a estaba **ausente**)
  - ○ 18e. En el baño
  - ○ 18f. En la clase de educación física, en los casilleros o duchas del gimnasio
  - ○ 18g. En el casino/comedor de la escuela
  - ○ 18h. De ida y vuelta a la escuela
  - ○ 18i. En la parada de la liebre o bus escolar
  - ○ 18j. Dentro de la liebre o bus escolar
  - ○ 18k. En alguna otra parte de la escuela

19. ¿Le has **contado a alguien** que has sufrido bullying en la escuela en los últimos dos meses?
  - ○ No he sufrido bullying en la escuela en los últimos dos meses
  - ○ Me han hecho bullying, **pero no se lo he contado a nadie**
  - ○ Me han hecho bullying **y se lo he contado a alguien**

  Rellena los círculos que correspondan a todas las personas a las que les has contado:

  - ○ 18a. A tu profesor/a
  - ○ 18b. A otro adulto en la escuela
  - ○ 18c. A tu papá, mamá, apoderado/a
  - ○ 18d. A tu/s hermano/s o hermana/s
  - ○ 18e. A tu amigo/s o amiga/s
  - ○ 18f. A otra persona

20. ¿Qué tan frecuentemente **los profesores u otros adultos en la escuela** tratan de detener a un estudiante cuando le hace bullying otro?
    - ○ Casi nunca
    - ○ De vez en cuando
    - ○ A veces
    - ○ Frecuentemente
    - ○ Casi siempre

21. ¿Qué tan frecuentemente **otros estudiantes** tratan de detener a un estudiante cuando le hace bullying a otro?
    - ○ Casi nunca
    - ○ De vez en cuando
    - ○ A veces
    - ○ Frecuentemente
    - ○ Casi siempre

22. ¿Algún **adulto de tu casa** se ha comunicado con la escuela en los últimos dos meses para pedir que otros estudiantes dejen de hacerte bullying?
    - ○ No he sufrido bullying en la escuela en los últimos dos meses
    - ○ No, no se han comunicado con la escuela
    - ○ Sí, se comunicaron con la escuela una vez
    - ○ Sí, se comunicaron con la escuela varias veces

23. Cuando presencias en la escuela que le están haciendo bullying a un estudiante de tu edad, ¿**qué piensas o sientes**?
    - ○ Que probablemente se lo merece
    - ○ No me afecta mucho
    - ○ Siento un poco de lástima por él o ella
    - ○ Siento lástima por él o ella y quisiera ayudarle

Cuando un estudiante le hace bullying a otros

24. ¿Qué tan frecuentemente has participado en actos de bullying contra un estudiante/s en la escuela **en los últimos dos meses**?
    - ○ No le he hecho bullying a otro/s estudiante/s en la escuela en los últimos dos meses
    - ○ Sólo ha ocurrido una o dos veces
    - ○ 2 o 3 veces al mes
    - ○ Aproximadamente una vez a la semana
    - ○ Varias veces a la semana

¿Le has hecho bullying a otro/s estudiante/s en la escuela en los últimos dos meses de alguna de las siguientes maneras? (preguntas 25-33)

Recuerda que el bullying es cuando un estudiante hacer sentir mal a otro a propósito y repetidamente.

25. Le puse apodos ofensivos, me burlé y lo/a molesté de forma hiriente.
    - ○ No ha ocurrido en los últimos dos meses
    - ○ Sólo una o dos veces
    - ○ 2 o 3 veces
    - ○ Aproximadamente una vez a la semana
    - ○ Varias veces a la semana

26. Lo/a excluí a propósito de lo que estaba haciendo, de mi grupo de amigos/as o lo/a ignoré por completo.
    - ○ No ha ocurrido en los últimos dos meses
    - ○ Sólo una o dos veces
    - ○ 2 o 3 veces
    - ○ Aproximadamente una vez a la semana
    - ○ Varias veces a la semana

27. Le pegué, pateé, empujé o lo/a encerré dentro de la sala de clases.
    - ○ No ha ocurrido en los últimos dos meses
    - ○ Sólo una o dos veces
    - ○ 2 o 3 veces
    - ○ Aproximadamente una vez a la semana
    - ○ Varias veces a la semana

28. Propagué rumores falsos de él o ella para provocar que les cayera mal a otros.
    - ○ No ha ocurrido en los últimos dos meses
    - ○ Sólo una o dos veces
    - ○ 2 o 3 veces
    - ○ Aproximadamente una vez a la semana
    - ○ Varias veces a la semana

29. Le quité dinero u otras cosas a él o ella o dañé sus pertenencias.
- ○ No ha ocurrido en los últimos dos meses
- ○ Sólo una o dos veces
- ○ 2 o 3 veces
- ○ Aproximadamente una vez a la semana
- ○ Varias veces a la semana

30. Lo/a amenacé o forcé a hacer cosas que él o ella no quería hacer.
- ○ No ha ocurrido en los últimos dos meses
- ○ Sólo una o dos veces
- ○ 2 o 3 veces
- ○ Aproximadamente una vez a la semana
- ○ Varias veces a la semana

31. Lo/a insulté con comentarios despectivos acerca de su país de origen, raza o color de piel.
- ○ No ha ocurrido en los últimos dos meses
- ○ Sólo una o dos veces
- ○ 2 o 3 veces
- ○ Aproximadamente una vez a la semana
- ○ Varias veces a la semana

32. Lo/a insulté con comentarios o gestos con tonos sexuales.
- ○ No ha ocurrido en los últimos dos meses
- ○ Sólo una o dos veces
- ○ 2 o 3 veces
- ○ Aproximadamente una vez a la semana
- ○ Varias veces a la semana

33a. Le hice bullying a otro/s mandando mensajes crueles e hirientes o fotografías ofensivas por medio de un celular o por Internet (computadora).

- ○ No ha ocurrido en los últimos dos meses
- ○ Sólo una o dos veces
- ○ 2 o 3 veces
- ○ Aproximadamente una vez a la semana
- ○ Varias veces a la semana

33b. Si le hiciste bullying a un/a estudiante usando un celular o por medio del Internet (computadora), ¿cómo lo hiciste?

- ○ Sólo por medio de un celular
- ○ Sólo por medio del Internet (computadora)
- ○ De las dos maneras

34. Le hice bullying de otra manera.
- ○ No ha ocurrido en los últimos dos meses
- ○ Sólo una o dos veces
- ○ 2 o 3 veces
- ○ Aproximadamente una vez a la semana
- ○ Varias veces a la semana

35. ¿Alguno/a de tus **profesores/as** ha hablado contigo en los últimos dos meses respecto a tu conducta de bullying hacia los demás?
   - ○ No le he hecho bullying a otro/s estudiante/s en la escuela en los últimos dos meses
   - ○ No, no han hablado conmigo
   - ○ Sí, hablaron conmigo una vez
   - ○ Sí, hablaron conmigo varias veces

36. ¿**Algún adulto de tu casa** ha hablado contigo en los últimos dos meses respecto a tu conducta de bullying hacia los demás?
   - ○ No le he bullying a otro/s estudiante/s en la escuela en los últimos dos meses
   - ○ No, no han hablado conmigo
   - ○ Sí, hablaron conmigo una vez
   - ○ Sí, hablaron conmigo varias veces

37. ¿Si le están haciendo bullying a un estudiante que te cae mal, crees que tu participarías también?
   - ○ Sí
   - ○ Sí, quizás
   - ○ No sé
   - ○ No, no creo
   - ○ No
   - ○ Definitivamente no

38. ¿Por lo general, **qué haces** si ves o te enteras que otros estudiantes le están haciendo bullying a un estudiante de tu edad?
   - ○ Nunca he visto que le hagan bullying a un estudiante de mi edad
   - ○ Tomo parte del bullying también
   - ○ No tomo parte del bullying, pero creo que no tiene nada de malo
   - ○ Sólo observo lo que pasa
   - ○ No hago nada, pero creo que debería ayudar
   - ○ Trato de ayudar al estudiante de una forma u otra

39. ¿Qué tan frecuentemente tienes miedo de que otros estudiantes te hagan bullying?
   - ○ Nunca
   - ○ Rara vez
   - ○ A veces
   - ○ Con cierta frecuencia
   - ○ Con frecuencia
   - ○ Con mucha frecuencia

40. ¿Cuánto crees que **tu profesor/a** ha hecho en los últimos dos meses para disminuir los actos de bullying entre sus estudiantes?
   - ○ Poco o nada
   - ○ Muy poco
   - ○ Algo
   - ○ Bastante
   - ○ Mucho

41. Según tu opinión, ¿qué piensa tu profesor/a acerca del bullying?

- ○ Piensa que el bullying es algo bueno
- ○ No le importa si los estudiantes sufren bullying o no
- ○ No lo sé
- ○ Cree que el bullying está mal
- ○ Cree que el bullying es algo inaceptable

42. ¿Qué piensan tus padres o apoderados acerca del bullying?

- ○ Piensan que el bullying es algo bueno
- ○ No les importa si los niños sufren bullying o no
- ○ No lo sé
- ○ Piensan que e bullying está mal
- ○ Piensan que el bullying es algo inaceptable

B. A continuación encontrarás un cuadro donde aparecen afirmaciones sobre cómo es tu vida en la escuela, específicamente cómo te sientes ahí. Contesta frente a cada afirmación rellenando el círculo junto a la respuesta que mejor describa lo que piensas o sientes, teniendo en cuenta si estás de acuerdo o en desacuerdo con las afirmaciones. **Sólo rellena un círculo por cada afirmación.**

¿Hasta qué punto estás de acuerdo o en desacuerdo?

0 = Completamente en desacuerdo
1 = Algo en desacuerdo
2 = No lo sé
3 = Algo de acuerdo
4 = Completamente de acuerdo

Completamente en desacuerdo <- - - - - - - - - - -> Completamente de acuerdo

	0	1	2	3	4
1. Me siento seguro/a en la escuela	○	○	○	○	○
2. Es habitual ayudar a los demás en nuestra clase	○	○	○	○	○
3. Estoy contento/a de estar en mi clase	○	○	○	○	○
4. Me siento aceptado/a cuando estoy en la escuela	○	○	○	○	○
5. Me gusta el ambiente en la escuela	○	○	○	○	○
